# Differences in dogs’ event-related potentials in response to human and dog vocal stimuli; a non-invasive study

**DOI:** 10.1098/rsos.211769

**Published:** 2022-04-06

**Authors:** Anna Bálint, Huba Eleőd, Lilla Magyari, Anna Kis, Márta Gácsi

**Affiliations:** ^1^ MTA-ELTE Comparative Ethology Research Group, Budapest, Hungary; ^2^ Department of Ethology, ELTE Eötvös Loránd University, Budapest, Hungary; ^3^ Doctoral School of Biology, Institute of Biology, ELTE Eötvös Loránd University, Budapest, Hungary; ^4^ MTA-ELTE ‘Lendület’ Neuroethology of Communication Research Group, Hungarian Academy of Sciences, ELTE Eötvös Loránd University, Budapest, Hungary; ^5^ Department of Social Studies, University of Stavanger, Stavanger, Norway; ^6^ Institute of Cognitive Neuroscience and Psychology, Research Centre for Natural Sciences,Budapest, Hungary

**Keywords:** dog, non-invasive ERP, acoustic stimuli, valence, species differentiation

## Abstract

Recent advances in the field of canine neuro-cognition allow for the non-invasive research of brain mechanisms in family dogs. Considering the striking similarities between dog's and human (infant)'s socio-cognition at the behavioural level, both similarities and differences in neural background can be of particular relevance. The current study investigates brain responses of *n* = 17 family dogs to human and conspecific emotional vocalizations using a fully non-invasive event-related potential (ERP) paradigm. We found that similarly to humans, dogs show a differential ERP response depending on the species of the caller, demonstrated by a more positive ERP response to human vocalizations compared to dog vocalizations in a time window between 250 and 650 ms after stimulus onset. A later time window between 800 and 900 ms also revealed a valence-sensitive ERP response in interaction with the species of the caller. Our results are, to our knowledge, the first ERP evidence to show the species sensitivity of vocal neural processing in dogs along with indications of valence sensitive processes in later post-stimulus time periods.

## 1. Introduction

Natural vocalizations have the potential to convey a variety of different information both in humans and non-human animals. This notion is also supported by the conspicuous and shared finding across a wide range of vertebrate species that certain brain regions prefer vocalizations over other types of sound stimuli (e.g. amphibians, reptiles, birds, rodents, carnivores, primates; for a review see [1]. The conveyed information may range from supraindividual characteristics such as group or species membership to various individual features such as age, sex or the emotional/motivational state of the caller [1–4]. Two widely studied characteristics are the perception of species membership and emotional state, as demonstrated by a large number of studies exploring both the encoding and perception of such information (for a review see [1]). Undoubtedly, the accurate decoding of these features is advantageous for the listener in adjusting their behaviour accordingly and managing social interactions. Because of the closer physical and often social association of species living in mixed-species groups, deciphering such cues may be of particular importance in forming and maintaining social bonds or acquiring relevant environmental information (e.g. alarm calls). Indeed, interspecies communication has been described in a number of mammalian and bird species [5,6]. In this regard, investigating the vocal processing of conspecificity and emotionality in dogs is particularly interesting. Dogs have become a part of the human social environment over the course of domestication, and most of them interact with both humans and other dogs on a regular basis, plausibly suggesting that they have become adept at navigating themselves in both con-, and heterospecific vocal interactions. Accordingly, behavioural studies have shown that dogs can match humans' [7,8] and dogs’ pictures with their vocalizations [8], as well as dog and human emotional vocalizations with the congruent facial expressions [9]. In recent years, dogs have also become an increasingly popular model species of comparative neuroscience owing to several different factors. These include the above-discussed and other functional behavioural analogies between dogs and humans (for a review see: [10], dogs' cooperativeness, trainability [11] and a recent advance in non-invasive neuroscientific research methodologies in dogs, for example, functional magnetic resonance imaging (fMRI) [12,13], polysomnography (e.g. [14–17] and event-related potentials (ERPs) [18,19]). However, behavioural analogies do not necessarily mean the same underlying neurocognitive processes (e.g. [12,15,16]), thus investigating the neural processes in parallel to behavioural observations is most certainly needful (e.g. [20]). Therefore, in the present study, our aim was to explore the neural processing of emotionally loaded con-, and heterospecific vocalizations in dogs by investigating the temporal resolution of these processes for the first time, to our knowledge, in an ERP experiment.

In general, the neural processing of conspecificity has been found to show similarities across mammalian taxa as demonstrated by different neurophysiological (e.g. [21,22]) and neuroimaging studies revealing voice-sensitive brain areas in several different species (e.g. marmosets [23]; dogs [13]; humans [24]). ERP evidence for the special processing of voiceness has also been found. While the majority of such studies have been conducted in humans (e.g. [25,26]), non-human animals have also been investigated more recently (e.g. horses: [27]). There is variation in the appearance and distribution of the voice-related components in humans depending partly on the electrode site and differing between studies as well. For example, some studies have found one prominent time-window showing voice specificity (e.g. 60–300 ms [26]); 260–380 ms [28]), while other studies have found several, sometimes overlapping time periods (e.g. 74–300 ms, 120–400 ms 164–400 ms [29]; 66–240 ms, 280–380 ms [30]). It is also important to note that although the processing of conspecific vocalizations—at least in part—seems to be based on innate capacities, early experience and learning can also play a major role as has been shown e.g. in songbirds [31].

Considering the neural processing of emotional vocalizations, a large body of behavioural experimental evidence indicates that there is differential hemispheric involvement in emotional processing. In general, most studied vertebrate species—including dogs [32]—show a right-hemispheric bias for negatively connotated emotions while a left hemispheric bias for positively connotated emotions (see [33]). In humans, there is also a large number of more direct neural investigations on emotional processing, including both fMRI and ERP studies. For instance, certain brain regions (e.g. parts of the auditory cortex, amygdala, medial prefrontal cortex) are more active for positive and negative sound stimuli than for neutral ones [34,35] and several different ERP components have been linked to emotional processing from early components such as N1, P2 (e.g. [36]), early posterior negativity [37] to later components as the late positive potential (LPP; [38]). Although similar neural evidence is much scarcer in non-human animals, there are indications that some brain mechanisms involved in emotional processing are similar across certain species (e.g. involvement of the amygdala in rats: [39]; bats: [40]; primates: [41]; humans: [42]). Additionally, since the vocal expression of emotions shows a remarkable similarity in its acoustic properties across mammalian species (for a review see [1]), the decoding of emotional information may even function between species (e.g. [43]).

In the present study, we tested family dogs—previously trained to lie motionless for up to 7 min—in a passive listening experimental paradigm while their electroencephalogram (EEG) was measured. The stimuli used in the study included both non-verbal human and dog vocalizations, similar to the ones used in the comparative fMRI study of Andics *et al*. [13], ranging from neutral to positively valenced sounds (as rated by human listeners, see [43]). In Andics *et al*. [13], they have found conspecific preferring regions in both dogs and humans, as well as similar near-primary auditory regions associated with the processing of emotional valence in vocalizations. These regions responded stronger to more positive valence and interestingly, overlapped for conspecific and heterospecific sounds in both species. However, there is little known about the temporal processing of such stimuli in dogs. We hypothesized that similarly to previous behavioural and neuroimaging studies, we may also find differential ERP responses in dogs depending on the species of the caller and/or the emotional content of the stimuli. We were also interested in whether these effects will have a similar temporal trajectory to the processing of such stimuli described in human ERP literature.

## 2. Methods

### 2.1. Subjects

We tested 24 family dogs, but seven dogs were excluded owing to the low number of trials left after the artefact rejection procedure. Thus, we included 17 subjects in our final analyses (nine males, eight females; age: 2 to 12 years (mean = 5.1 years); three border collies, two golden retrievers, two labradoodles, two Australian shepherds, two English cocker spaniels, one Hovawart, one Cairn terrier, one Tervueren, one German shepherd and two mixed breeds). All dogs were trained to lie motionless for extended durations according to the method described in Andics *et al*. [13].

### 2.2. Electrophysiological recordings

The electrophysiological recordings were carried out according to the completely non-invasive polysomnography method developed and validated by Kis *et al*. [14] and applied in many studies since (e.g. [15,44,45]). According to the procedure, we recorded the EEG (including electrodes next to the eyes, used as eletrooculogram (EOG); mainly for detecting artefactual muscle movements), electrocardiogram and the respiratory signal of dogs, but only used the EEG signal in these analyses.

**Figure 1.**
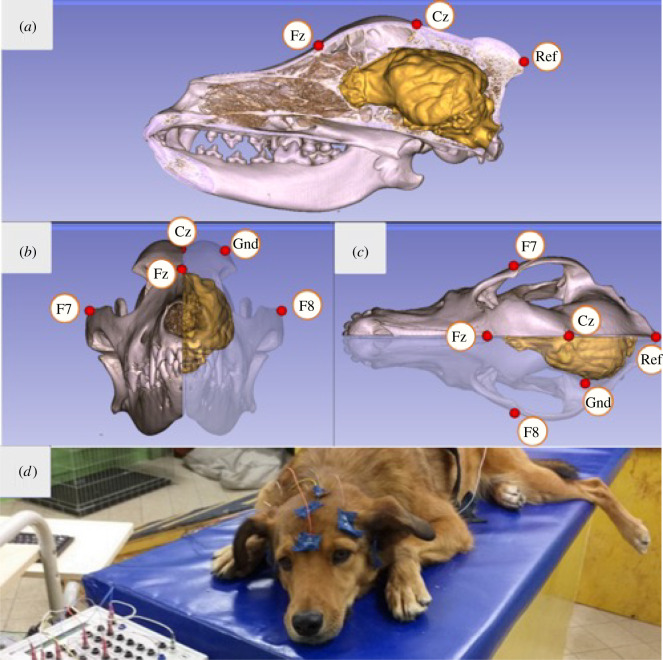
Positions of the Fz and Cz electrodes relative to the three-dimensional model and endocranial cast of the skull of a pointer dog (with yellow showing the brain's morphology): (*a*) lateral, (*b*) anterior and (*c*) superior views, image courtesy of Kálmán Czeibert. (*d*) Photograph showing a dog during the measurement. A video showing the electrode placement procedure can be found at: https://youtu.be/OYc7ALKtowk.

Surface attached, gold-coated Ag/AgCl electrodes were used, fixed to the skin by EC2 Grass Electrode Cream (Grass Technologies, USA). Two electrodes were placed on the frontal and central positions of the anteroposterior midline of the skull (Fz, Cz) and two electrodes on the right and left zygomatic arch, next to the eyes (EOG: F7, F8), all positioned on bony parts of the dogs' head, in order to reduce the number of possible artefacts caused by muscle movements. All four derivations were referred to an electrode at the posterior midline of the skull (Ref; occiput/external occipital protuberance), while the ground electrode (Gnd) was placed on the left musculus temporalis (figure 1). Impedance values were kept below 20 kΩ.

The signals were amplified by a 40-channel NuAmps amplifier (© 2018 Compumedics Neuroscan) and digitized at a sampling rate of 1000 Hz/channel, applying DC-recording.

### 2.3. Experimental set-up

**Figure 2.**
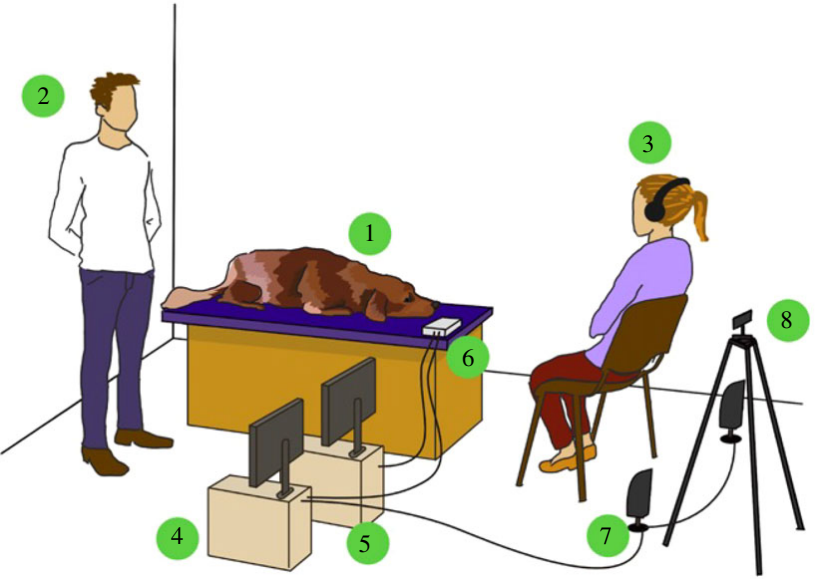
The general set-up of the experiment 1. Dog subject; 2. experimenter; 3. owner/trainer; 4. computer: presenting the stimuli; 5. computer: recording the signal; 6. EEG amplifier; 7. speakers; 8. mobile phone for video recording.

The experiments were conducted in a 5 × 6 m laboratory fully equipped for neurophysiological measurements at the Department of Ethology, University of ELTE. The dogs were lying on a 1.5 m high wooden, cushioned platform during the experiment. A computer recording the EEG signal and a computer controlling the stimuli were located next to the platform. The EEG amplifier was placed on the platform, next to the dog's head. In front of the platform, there were two speakers emitting the acoustic stimuli (Logitech X-120 speakers, 1 m in front of the platform and 1 m apart from each other) and a camera (Samsung Galaxy J4 + mobile telephone, 1.5 m in front of the platform) recording the dog during the experiment. Two people were present during the experiments, the experimenter and a familiar person (mostly the owner, but if the dog was newly trained, the dog's trainer was present). The experimenter stood behind the dog (out of the dog's sight) throughout the experiment, while the owner/trainer remained in front of the dog (figure 2).

### 2.4. Stimuli

The acoustic stimuli consisted of non-verbal vocalizations collected from dogs and humans, recorded and analysed in a previous study by Faragó *et al*. [43]. In that study, human subjects were asked to rate 100 human and 100 dog vocalizations along two dimensions, emotional valence and emotional intensity [43]. In the current study, we used 10 sound samples with the highest emotional valence scores (positive) and 10 samples with lowest absolute value scores (neutral) from both the human and dog vocalizations resulting in 20 stimuli from both species. There were four types of stimuli: positive-dog (PD); neutral-dog (ND); positive-human (PH); neutral-human (NH), consisting of sniffing, panting, barking in the case of dog vocalizations and yawning, laughter, coughing and infant babble in the case of human vocalizations. The duration of all sound-files was equal (1 s), and the volume of the sound-files did not differ across conditions (one-way ANOVA: *M*(all) = 69.75 dB, s.d. = 1.51, *F*_1_ = 0.16, *p* = 0.70). One recording session consisted of 32 stimuli (eight sound samples from each condition), played back in a semi-random order (less than three sounds from the same type could follow each other) with jittered interstimulus intervals (9 to 15 s).

### 2.5. Experimental procedure

Upon arrival, the experimenter outlined the course of the experiment to the owner while the dog was allowed to freely explore the room (5–10 min). The dog was then asked to ascend the platform on a ramp and lie down, facing the owner (or the trainer). After the dog settled, the experimenter attached the electrodes to the dog's head, carefully checking the signal quality and impedance values before signal acquisition. If the visual inspection of the EEG showed a clear signal and impedance values on all electrodes were below 20 k*Ω*, the experimenter assumed a position next to the platform (in front of the recording computer and out of the dogs' sight) and started both the signal acquisition and stimulus playback (synchronized to each other). In order to avoid the influence of unintentional responses from the owner to the acoustic stimuli, the owners (or trainer) were wearing headphones to block out the stimuli and were also asked to avoid maintaining direct eye contact with the dog. The owner (or trainer) remained in front of the dog, ensuring it remained motionless throughout the experiment by using hand gestures and nonverbal communication, should it be necessary. If the owner/experimenter considered the dog to be tired, the recordings were ended for that day. The trials affected by the movements were rejected during the artefact-rejection process.

Each dog had several recording occasions (2 to 6 occasions, mean = 3.8 ± 1.2) on different days and several recording sessions on each occasion (2 to 4 sessions, mean = 2.6 ± 0.6) depending on the dog's training status and level of tiredness, assessed by the owner or trainer. One session lasted 6 to 7 min, depending on the varying length of inter-stimulus intervals. Between the sessions, the dog was rewarded and was allowed to move around freely in the laboratory. Summing up all occasions, the dogs participated in 6 to 12 sessions (mean = 9.6 ± 2.2; the high variance being due to the different amount of artefacts in each subject).

### 2.6. Analytical procedures

We segmented our data in two different post- (and pre-) stimulus time intervals. For the main analysis, we used segments from 200 ms before to 1000 ms after the onset of the stimuli. For the extended analysis, we used segments from 200 ms before to 2000 ms after the onset of the stimuli. The extended analysis was done in order to explore possible late, post stimulus-offset effects, therefore we only analysed the time-segment between 1000 and 2000 ms in this analysis [46,47]. As the two analyses were handled separately, the corresponding data preprocessing and artefact rejection processes were also somewhat different in the two cases (see in later paragraphs).

In order to compare our results with the findings of human ERP studies, we first analysed our data in time-windows corresponding to ERP components found in the literature of voice and vocal emotion processing in humans in both the main and in the extended analysis (literature-based time-windows). Additionally, since the potentials recorded in different species can be different for several different reasons including head size, axonal path lengths, gyrification patterns or the specific auditory cell types [48], we also conducted an exploratory, overlapping sliding time-window analysis on our data to more precisely evaluate the on- and offset times of possible effects (as in [19]) in both the main and in the extended analysis (sliding time-window analysis).

For the literature-based time-window analysis, the selection of relevant time windows for statistical analysis was based on the human literature. A number of different components have been linked both to voice and vocal emotion processing. However, the exact timing of these components shows a huge variety between different studies depending on the study design, stimulus characteristics and task requirements. Therefore, we selected time windows from the literature that appeared to be the most applicable to our study. Interestingly, although with slightly different or overlapping time windows, mostly similar components have been implicated both in voice and vocal emotion processing. In the main analysis, the earliest of these components linked both to voice and emotion effects is the N100: 80–120 ms [49]), then the P200. We selected, therefore, the N100 (80–120 ms) and a P200 window. Because different studies assign different time windows to the P200 depending on its voice or emotion sensitivity, we selected a window including time-periods linked to both: 150–350 ms (voice/emotion: ‘P2/P3’: 150–350 ms [49]; emotion: 150–300 ms [50]). Another selected time window was the window of the P300 component which has been linked to emotion processing in the human EEG literature: 250–400 ms [51]. The next affected component is the LPP, an extending positivity beyond the P300. While it has widely been described as a robust marker of emotionally loaded stimuli from various modalities, some studies have also found it to be modulated by voice, although in interaction with emotional content [49]. Therefore, we selected more than one time window for this component, depending on its emotion or voice sensitivity (emotion sensitive time window: 450–700 ms [52,53]; voice/emotion sensitive time window: 500–800 ms [49]). In the extended analysis, we based our time-window selection on results suggesting that the LPP component or the effects of other relevant stimulus features (e.g. visual symmetry: [47]) may even extend to post-stimulus-offset time periods [46,54]. Therefore, to investigate possible late ERP modulation effects manifesting after the completion of the stimuli, we examined a 1 s long time-period after the offset of the stimulus from 1000 to 2000 ms (as in [54]) in this analysis.

In the exploratory sliding time-window analysis, we systematically analysed the EEG data by performing a 50 ms consecutive time-window analysis on the segments of the main and the extended analysis averaged for each dog. In the main analysis, the interval from 0 to 1000 ms (0–1000 ms) was analysed, while in the extended analysis, the interval from 1000 ms to 2000 ms was analysed with 100 ms long overlapping windows (between 0 and 100 ms, 50 and 150 ms, 100 and 200 ms etc., as in [19].

### 2.7. Preprocessing and artefact rejection

EEG preprocessing and artefact rejection were done using the FieldTrip software package [55] in Matlab 2014b. First, the continuous EEG recording was filtered using a 0.01 Hz high-pass and a 40 Hz low-pass filter. The data were then segmented into 1200 ms long trials in the main analysis and 2200 ms long trials in the extended analysis, with a 200 ms long pre-stimulus and a 1000 ms (or 2000 ms) long interval after the onset of the stimulus. Each trial was detrended (removing linear trends) and baselined (using the 200 ms long pre-stimulus interval).

The artefact-rejection process of the main analysis consisted of three consecutive steps, following the methodology outlined by Magyari *et al*. [19]. The trials of each subject were first subjected to an automatic rejection process, excluding all trials with amplitudes exceeding ±150 µV and differences between minimum and maximum amplitude values exceeding 150 µV in 100 ms sliding windows (automatic rejection). Next, the videos recorded during the experiments were annotated according to the stimulus onsets using the ELAN software [56], selecting video-clips between 200 ms before and 1000 ms after the stimulus onset for every trial remaining after the automatic rejection phase. These video-clips were then visually evaluated and trials containing any movement (apart from breathing movements) were excluded (video rejection). Third, the remaining trials were visually inspected for residual artefacts (visual rejection). In order to more precisely identify eye movements, additional bipolar derivations were created: a horizontal ocular channel using the F7 and F8 channels (F7F8), and by referring the eye derivations to Fz (F7Fz; F8Fz). The artefact rejection process of the extended analysis was performed on the trials remaining after the video rejection step of the main analysis' artefact rejection process. It consisted of only two steps, an automatic rejection and a visual rejection step, with the same parameters as described earlier, but owing to the longer segments (2000 ms instead of 1000 ms) more trials were excluded during these phases.

Visual inspection of the video-clips and the visual rejection step was done by one of the authors (H.E.) with a subset of trials (video rejection: *n* = 594; visual rejection: *n* = 250) being inspected by an additional person (A.B.)—both blind to the experimental conditions—in order to control for coding reliability. Interrater reliability tests (performed using IBM's SPSS software (https://www.ibm.com/products/spss-statistics), showed a substantial agreement (according to the categorization by [57] between observers with the Cohen Kappa value of 0.724 in the case of the video-clip evaluation and a Cohen Kappa value of 0.736 in the case of visual rejection (including trials from both the main analysis' and extended analysis' visual rejection step)) (table 1).

**Table 1.** Percentage of excluded trials in the three phases of the artefact rejection process in the main and extended analyses. PD: positive dog, ND: neutral dog, PH: positive human, NH: neutral human.

	condition	rejected trials (%) in the three phases of the artefact rejection process			remaining trials (%)
		automatic	video	visual	
main analysis (0–1 s)	PD	20.34	44.63	7.18	27.85
	ND	17.33	46.44	6.88	29.35
	PH	19.63	48.11	6.44	25.82
	NH	19.63	47.60	5.61	27.16
	overall	19.22	46.70	6.53	27.55
extended analysis (1–2 s)	PD	34.18	35.73	13.62	16.48
	ND	32.17	37.10	12.02	18.71
	PH	36.70	35.72	11.47	16.11
	NH	34.11	37.50	11.37	17.02
	overall	34.28	36.52	12.12	17.09

Based on trial numbers used in infant studies (e.g. [58,59]), we excluded subjects from the main analysis if less than 15 trials were left in any of the conditions after the artefact rejection process (with two exceptions: one subject had 14, another had 12 trials in one condition, while more than 15 trials in all other conditions). In the case of the extended analysis, the threshold was lowered to five trials (in one case four trials) to avoid losing subjects from our analyses. At the same time, we set *n* = 100 as the upper limit to the number of trials per subject, in order to maintain a relatively low standard deviation in our dataset. Our final dataset contained 80.9 ± 8.6 trials per subject (PD = 20.3 ± 3.9, ND = 21.9 ± 2.7, PH = 18.9 ± 3.3, NH = 19.9 ± 4.2) in the main analysis, which decreased to 49.8 ± 10.5 trials per subject (PD = 11.9 ± 3.9, ND = 13.8 ± 3.5, PH = 11.6 ± 3.3, NH = 12.4 ± 3.5) in the extended analysis.

### 2.8. Statistical analysis

In the statistical models, we tested how the evoked potentials are modulated by the species of the caller, the valence of the sound and by the electrode site. We performed linear mixed model (LMM) analyses in R [60]. We selected the best fitting model by comparing the Akaike information criterion score of potential models using a top-down approach with backward elimination. The best fitting model consisted of the three main factors: species, valence and electrode, the interaction of species and valence and an additional random slope of valence. In the literature-based time-windows analyses, the data entered into the models were the averaged ERP values of each subject in the given time windows. In the sliding time-window analyses, the data entered were the average EEG values of each subject in the corresponding 100 ms long time window. Consecutive 100 ms time windows showing a statistically significant ERP modulation effect were further analysed as a single, conjoined window. Although the two electrode sites measured (Fz and Cz, see Methods) in our current experimental design are far less than the number of electrodes used in humans, they still hold the potential for some level of anterior–posterior differentiation between measurements, rendering them relevant as model factors. Detailed statistical results are shown in the electronic supplementary material, tables S1 and S2.

## 3. Results

### 3.1. Literature-based time-windows

#### 3.1.1. Main analysis

N100 (80–120 ms); P200 (150–350 ms); LPP (500–800 ms): we have found no statistically significant effects in this time-window.

**Figure 3.**
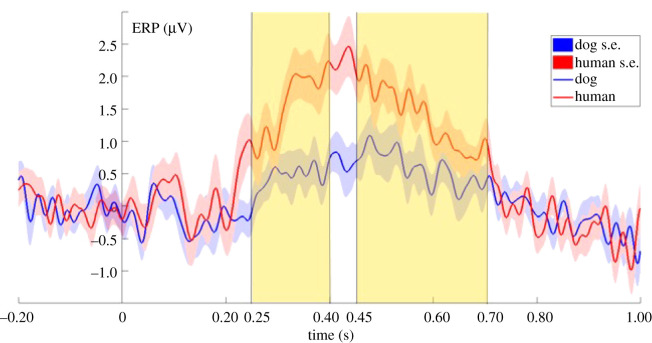
Grand-averaged ERPs showing the two averaged levels of the species factor, from 200 ms before to 1000 ms after stimulus onset (0 point on the *x*-axis). The highlighted parts show the time windows between 250 ms to 400 ms and 450 ms to 700 ms where the species effect was significant in the literature-based time-windows analysis.

P300 (250–400 ms): the ERP response was more positive to human than to dog sounds (LMM: *F*_1,99_ = 6.0497; *p* = 0.0156; figure 3), while the valence of the sounds had no significant effect on the ERP responses.

LPP (450–700 ms): the ERP response was more positive to human than to dog sounds. (LMM: *F*_1,99_ = 4.4397; *p* = 0.03764; figure 3), while the valence of the sounds had no significant effect on the ERP responses.

For detailed results, see the electronic supplementary material, table S1.

#### 3.1.2. Extended analysis

1000–2000 ms: we have found no significant effects in the post-stimulus-offset time window.

### 3.2. Sliding time-window analysis

#### 3.2.1. Main analysis

**Figure 4.**
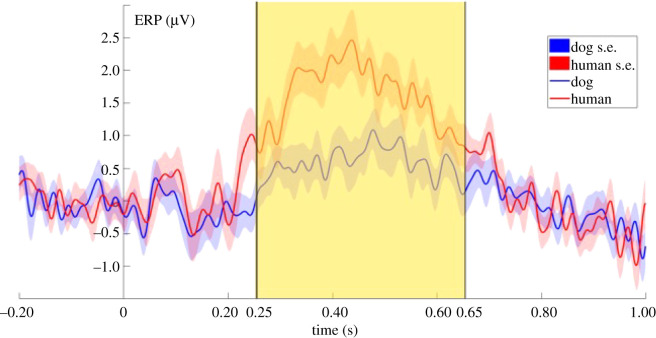
Grand-averaged ERPs showing the two averaged levels of the species factor, from 200 ms before to 1000 ms after stimulus onset (0 point on the *x*-axis). The highlighted part shows the time window between 250 and 650 ms where the species effect was significant in the sliding time-window analysis.

The sliding time-window analysis revealed seven consecutive 100 ms time windows (from 250 ms to 550 ms) showing a significant species main effect, constituting a time-window between 250–650 ms where dogs showed a more positive ERP response to human than to dog vocalizations (LMM: *F*_1,99_ = 6.9068; *p* = 0.00995; figure 4). In the same time window, the valence of the stimuli had no significant effect on the ERP responses.

We have also found six consecutive time windows (from 350 ms and 600 ms) showing a significant electrode main effect, with different ERP amplitudes at the Fz and Cz derivations between 350 and 700 ms (LMM: *F*_1,99_ = 7.8148; *p* = 0.006).

**Figure 5.**
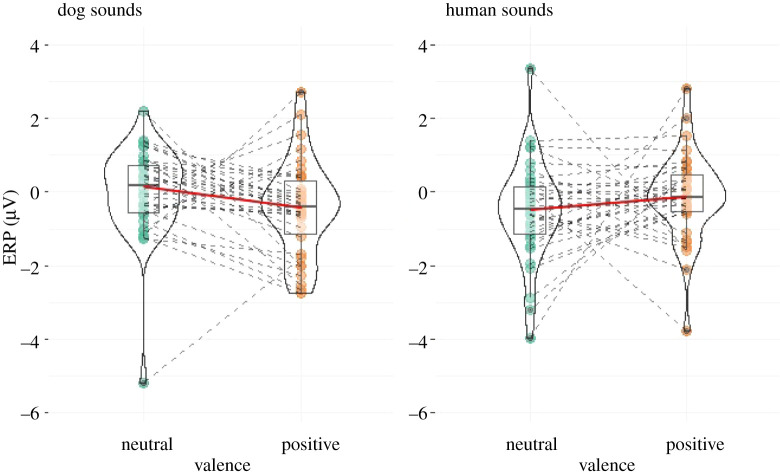
Species-valence interaction effect found in the time-window between 800 and 900 ms, registered ERPs shown in the four different conditions. Green and yellow dots connected with dashed lines indicate ERP values for each individual dog. The boxplots show the medians (connected with red lines), upper and lower quartiles and whiskers. The violin plot shows the probability density of the data at different values.

Finally, we have found one 100 ms window between 800 and 900 ms that revealed a significant effect for a species × valence interaction (LMM: *F*_1,99_ = 4.4128; *p* = 0.038). ERP responses to positive and neutral stimuli were different depending on the species of the caller: while responses were more positive to neutral stimuli in the case of dog vocalizations, they were more positive to positive stimuli in the case of human vocalizations (figure 5).

For the detailed results of the sliding time-window analysis see the electronic supplementary material, table S2.

#### 3.2.2. Extended analysis

In the extended analysis, we have found no significant effects in either of the 100 ms time windows.

### 3.3. Individual ERP responses

**Figure 6.**
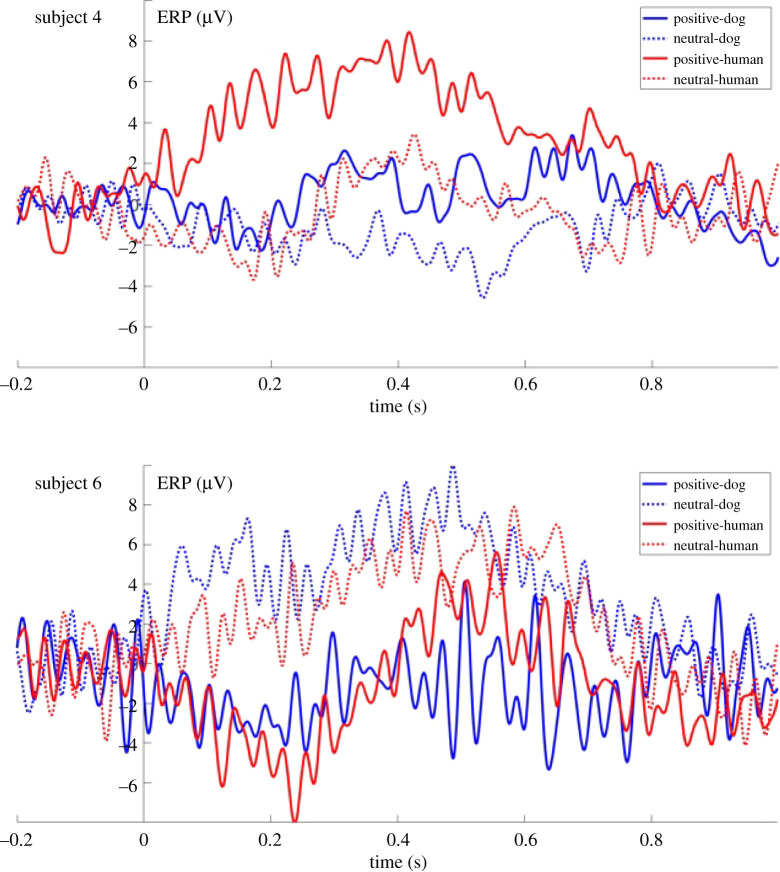
Individual ERP response of two subjects (subjects 4 and 6).

The visual inspection of the dogs' ERP responses (see the electronic supplementary material, figure S1) suggested that some subjects—instead of or in addition to the above-described species-dependent ERP response—show a valence-related or valencexspecies interaction related ERP modulation effect, seemingly differentiating between positive and neutral auditory stimuli. Although our experimental design and sample size do not allow us to reveal all underlying neural processes, the plots of all individual ERP results are presented in the electronic supplementary material, figure S1, to provide a comprehensive account of our results (figure 6).

## 4. Discussion

In this study, we investigated the temporal processing of emotionally valenced dog and human vocalizations in dogs, using ERP measurements. We have found species-effects in two time windows in the analysis which was based on a priori selected time windows. The sliding time-window analysis showed that the temporal borders of this effect are between 250 and 650 ms. In this time window, dogs showed a differential ERP response depending on the species of the caller by showing a more positive ERP response to human compared to dog vocalizations. Both the human and dog vocalizations caused a positive deflection in the EEG signal which is comparable to what other auditory ERP studies on dogs have found in response to sounds (such as words or beep stimuli) using the same [19] or a similarly localized reference electrode [61]. This time window coincides with two components known from the human ERP literature—the P300 and LPP—as demonstrated by our literature-based analyses. We have not found any significant effects in the other literature-based time-windows, nor in the extended analysis.

The direct comparison of ERP components between species is far from straightforward since there are a number of potential differences between species that can affect the appearance of an ERP wave, including the size of the brain, differences in the brain's folding pattern [48] or sensory thresholds [62]. Nevertheless, since there is a growing body of evidence showing analogies in the brain regions involved, or the neural processing mechanisms of auditory signals across a large number of species [1], the comparison of human ERP components and ERP waveforms found in other species is still warranted. The auditory N100 (80–120 ms) component has been linked to emotional processing in a number of studies [49,51,63], but only few have found it to be modulated by voiceness [49]. Many of these studies have corroborated the notion that the N100 is mostly sensitive to the acoustic properties of the sound stimuli [29,64] and thus reflects a coarse categorization of stimuli related either to emotional content or voiceness. Since we have not found any effect in this time range, we may hypothesize that although emotionally loaded sound stimuli from different species are inherently different in their acoustical parameters, in this set of stimuli this difference may not have been prominent enough to elicit a measurable difference in ERP responses. The time-period of the P200, with a latency beginning around 150 ms after stimulus onset has also been linked both to voice [29] and emotion processing [64]. It has been related to an early categorization of sounds in terms of its ‘voiceness’ [29] and has also been shown to be modulated by emotional quality of the sound as well as other stimulus properties such as pitch, intensity or arousal [64]. The lack of any effects in this time-window may once again point to the possibility that the acoustic ‘contrast’ between different stimuli (e.g. positive and neutral vocalizations) was not conspicuous enough to allow for rapid, early categorization of sounds. The following P300 (from 250 ms on) and LPP components (from 450 ms on) have both been shown to be increased by the emotional content of stimuli and the LPP has often been described as a series of overlapping positive deflections beginning with the P300 component, lasting for several hundred milliseconds [46,65–67]. Interestingly, however, our results revealed the significant effect of the species of the caller instead of the emotional valence of the sounds in these time periods. Importantly, the sensitivity of these components appears to be related to the motivational significance and salience of stimuli (also intrinsic to emotional stimuli), capturing attention automatically [38]. The LPP is suggested to reflect this sustained attention to motivationally significant stimuli, even withstanding habituation over repeated presentations of the same stimuli [37,68–70]. Thus, the extended difference in the ERP responses to human and dog vocalizations between 250 and 650 ms may reflect a difference between the motivational significance and thus the allocated attention to human and dog vocalizations. This difference may be explained by the very different roles that humans and other dogs play in the social life of dogs. These qualitatively different relations and the need to manage diverse types of social interactions may be reflected in the differential processing of dog and human vocal signals. Considering the underlying neural mechanism, the effect may also be owing to the different brain areas responsible for the processing of hetero- and conspecific vocalizations (as has been demonstrated by Andics *et al*. [13]).

Additionally, the sliding time-window analysis has revealed a significant interaction effect of the species and valence factors in an even later time window between 800 and 900 ms. Because later periods of an ERP waveform generally reflect higher-level cognitive processes [37,71], the sustained modulation effect of the species and the late, species-dependent evaluation of valence information suggest that these ERP responses were related to a more subtle, higher-level processing of the vocalizations.

Our findings may also be interpreted within the conceptual framework of different processing stages in the voice processing of humans (e.g. [2,29,49]). The first stage is considered to correspond to a low-level categorization of sounds (e.g. living/non-living) around 100 ms after stimulus onset. A subsequent stage involves the more detailed analysis of the signal's caller (e.g. voice/non-voice) around the onset of the P200 component, while a third stage would represent a more complex processing of sounds merging different sound characteristics, prioritizing the processing of more significant 'sound objects’ [49] over others. We may argue that the lack of early ERP responses signals the fact of all stimuli belonging to the same broad category of ‘living’, while the later sustained modulation effect (and even later interaction effect) correspond to a more refined, higher-level processing stage of the stimuli.

Our results are also comparable with the fMRI study of Andics *et al*. [13]. Although our ERP study design is not suitable for the quantified comparison of response strengths in different topographical locations as the fMRI study, we could identify a time window where the ERP responses to human and dog vocalization differed from each other, most probably signalling the divergent underlying processing of the two types of signals. Additionally, we have also found a time-window where the stimuli's emotional content modulated the subjects' ERP response in interaction with the species of the caller. These temporal findings complement the spatial information gained by the fMRI experiment, particularly in light of the highly similar stimuli used in this and the study of Andics *et al*. [13].

We have also found a significant electrode effect in the 350–700 ms time window. Although this time window overlaps with the species effect between 250 and 650 ms, since it was not found to be in interaction with any of the other model factors, we primarily consider it as an independent effect of electrode placement that needs further studies involving anatomical data. The Cz electrode is closer to the A1 reference electrode, thus it is expected that the signal on Cz derivation appears to be smaller. Furthermore, brain imaging (MRI) studies on dogs (e.g. [72]) suggest that the distance from the brain to the skull might differ between the anatomical points used in the current study for electrode placements. Another potential factor that might affect EEG signals electrode-wise is the ratio of *ventriculus,* bone and other tissues between the recording sites and the brain.

The lack of any findings in the extended analysis should not be a basis for any strong conclusions, since only a very low number of trials remained after the artefact rejection process, and this was probably lowering the signal-to-noise ratio too much for any effect to emerge. Additionally, although there are studies showing that emotionally loaded or other relevant signals may have ERP effects even after the offset of the stimulus [47,54], there are a number of reasons why finding meaningful ERP responses at longer latencies is difficult. In general, ERPs are difficult to measure in longer time-periods because of various reasons from slow voltage drifts of non-neural origin (e.g. skin potentials, small static charges) to stimulus offset effects [37].

The high level of individual variability suggested by the visual inspection of the individual ERP responses may seem surprising, but different reasons may play a role in this phenomenon. The variability of ERP waveforms is a well-known phenomenon in human ERP research as well and can be related to both anatomical differences (e.g. skull thickness, brain's folding pattern) and individual differences in cognitive processing [71]. The first type of variation usually affects early latency changes in the ERP, reflecting differences in the sensory processing, while differences in the cognitive processing are mostly reflected in later ERP changes [71]. The fact that dogs show a huge intra-species morphological variability—including the physical characteristics of the skull [73]—may further increase the large variety of individual ERP waveforms.

Limitations of our study include the relatively small sample size owing to methodological constraints and difficulties. Participating dogs were selected from a special subset of dogs who were pre-trained to lie motionless for several minutes. Additionally, owing to the anatomical characteristics of dogs (showing large individual differences), EEG signals are inherently heavily affected by muscle movements, even in an apparently immobile dog. Because of these effects, not only the overall sample size but the amount of data collected from one subject may also be limited owing signal artefacts. Another limitation may be the lack of negative stimuli in the sound repertoire. However, we wanted to avoid the potential strong aversive effects that negatively valenced stimuli could have had on dogs, who were supposed to lie motionless. Since the time-frame to present the stimuli was also limited (conforming to the capacity of dogs to lie still), we abided by the application of the one-sided (neutral-positive) representation of the valence dimension. Lastly, because the valence of the stimuli cannot be directly scored by dogs but only human listeners, there is an inherent human bias in the valence ratings. Nevertheless, in a study using the full range of the same stimuli we used here [13], it has been shown that the context-valence, in which the dog vocalizations were recorded, covaried with the human valence ratings of the sounds, suggesting that human ratings represent a reasonably good evaluation of the animal's affective state.

In summary, we have found that similarly to humans, dogs also show a differential ERP response depending on the species of the caller. To the best of our knowledge, this is the first ERP evidence to show the species sensitivity of the vocal neural processing in dogs. Our findings also represent a new contribution to the field of non-human ERP research. Although impacted with a number of technical and methodological difficulties (e.g. training of dogs, low number of electrodes, high volume of artefacts), we believe that it is a research field worth pursuing, as it adds new and meaningful information to the increasing number of other non-invasive neuroimaging and electromagnetic measures of neural activity in the dog. Furthermore, it opens up the possibility of widening the range of comparative data from different species, an invaluable tool in gaining a better understanding of the underlying mechanisms of cognitive processes.

## Data Availability

The datasets and scripts used in the study can be accessed on Dryad via the following link: https://doi.org/10.5061/dryad.5qfttdz6m [74].
